# Odontological Society of Pennsylvania

**Published:** 1893-02

**Authors:** 

**Affiliations:** Odontological Society of Pennsylvania


### ODONTOLOGICAL SOCIETY OF PENNSYLVANIA.

The regular meeting was held December 10, 1892, at the rooms of the Society, 1228 Walnut Street, Philadelphia, Dr. Louis Jack presiding. After the transaction of the usual routine business, Dr. Bonwill rose to a question of privilege, and this being granted, said:

"Professor Truman, in the discussion on my essay, 'Mechanics in Dentistry,' etc., remarked, 'It is no wonder Dr. Bonwill did not value his degree; he never earned it. If he had gone through the schools, day after day sitting on the hard benches, and had studied along with others, he would have appreciated the feeling all have as students and as co-laborers. He came into the profession through another door, and there I leave him.'

"First, not a word that I said was against the college that gave me my diploma nor any of its professors. I meant that I thought nothing of my sheepskin only so far as hanging it in my office. I laid it away for reference, and only brought it forth when the officers demanded it for registration. The Pennsylvania College of Dental Surgery offered a degree of D.D.S. to all practitioners of fifteen years standing if they would take out tickets for one course, undergo examination, and pay for diploma.

"After having studied medicine in Jefferson Medical College, 1864, Professor Buckingham offered to give me what was considered an honorary degree by simply paying for diploma and submitting to examination. I appreciate the diploma as I do the many medals I have.

"Now, all I ask of my old friend, Professor Truman, is to do what I know he will, that the world may know I did not 'get into the profession through another door,' as he has insinuated. If I am not worthy, then he should bear the blame.

"I beg pardon for bringing such a delicate subject before the Society, but, it being on the record, and as Professor Truman wrote me he would have cut it out had he known I did not hear him, I deem it best to reply. If he cannot do me justice, then I am unable to find one who will and can.

"I leave the matter now to Professor Truman and this deliberative body."

*Dr. Truman.*—In reply to Dr. Bonwill, I have only this to say, that my remarks upon the occasion referred to had allusion entirely to the feeling accompanying the reception of a diploma conferred after laboring assiduously for it, and that which follows the one conferred honorarily. It was in this sense that I used the expression, "It is no wonder Dr. Bonwill did not value his degree; he never earned it." The balance of the paragraph makes the meaning clear, and I am surprised that any other construction should be put upon it. As Dr. Bonwill regards it differently, I wish to repudiate here distinctly any idea at the time of alluding to his work in the profession. If I were in favor of conferring honorary degrees for professional services, I would have great difficulty in finding one more worthy or better qualified to receive it than my friend, Dr. Bonwill. These need no laudatory notice at my hands, nor could any one by a word lessen the great value of the results of his laborious life. I certainly would not be that one. Hence I am glad to be able to make an explanation, and trust it will be accepted in the spirit in which it is given.

The subject was then passed.

Dr. Edward C. Kirk read a short paper on "Crystal Mat Gold," followed by a discussion of the topic presented.

(For Dr. Kirk's paper, see page 97.)

DISCUSSION.

*Dr. Darby.*—I have had a great deal of experience in years past with Watt's crystal gold. When it was introduced I was one of the first to try it, but my experience has not been as favorable as that of Dr. Kirk. I have found difficulty in working it, except in large, open cavities, where I could insert it with a broad-faced instrument. In cases of that kind I have found it very satisfactory; but the use I have put it to more commonly than others is, after bringing my filling near the surface, to use this gold for finishing. There it has worked very nicely. I have never been in love with Watt's crystal gold, and when asked why I use it I am sometimes at a loss for an answer. I think one reason is, that Watt's crystal gold, being a preparation of crystals, is liable to deteriorate more rapidly when exposed to the air. It presents a greater surface, and the crystals absorb moisture. I think it deteriorates more rapidly than any other preparation of gold.

*Dr. Woodward.*—I have used it, but to a very limited extent. My experience with it is something like Dr. Kirk's. If the gold is first carried to place, and partially condensed, and then malleted with any good mallet, it becomes very dense, is very cohesive, and sets where you place it. As long as the cavity is a large one I get along very rapidly, but when it is small it does not work as satisfactorily. The ordinary cohesive gold under a mallet makes fillings that would be like wrought iron compared with crystal gold. If I wanted great strength in fillings I should use the ordinary forms of cohesive gold; but crystal mat is of great use for various forms and places; in filling undercuts, for instance, or in a molar in which there is a large cavity, with strong enamel, and it is undesirable to cut the enamel wall away. This can be filled entirely with crystal mat, and can be burnished with a small ball-burnisher. In starting cervical edges it is very satisfactory, and especially with a matrix, as it can be crowded in several layers, with a broad plugger, into place. There are uses for it, but I think we will have to get acquainted with it before we can tell what they will be.

Dr. Kirk here stated that it was only in the class of cases that Dr. Darby and Dr. Woodward had called attention to that he advocated its use.

*Dr. Truman.*—I have had a little experience with it, and, from my observation, it works exactly like all the crystal gold I have ever used. I like it very well for a certain class of cavities; but the question comes up always, when crystal gold is discussed, What is it, and what does it do? Crystal gold, whenever it reaches the wall, or border, can never, in my opinion, be packed exactly solid. That matter was settled twenty years ago. The Pennsylvania Association of Dental Surgeons, of Philadelphia, made extensive examinations of crystal and sponge gold. I then spent a great deal of time in microscopical examination, and came to the conclusion that it could not be properly packed against the wall, for the simple reason that the crystals slide one upon the other, and it seems impossible to secure perfect cohesion there. The result of its use was a constant complaint of leakage, that being ascribed to various reasons, but it was always considered as the action of the uncondensed crystals.

I have spent a great deal of time in former years in studying gold crystals, and the working of gold preparations, rolled gold, rolled tin, and other materials, and this labor satisfied me that we know very little about the action of crystals. Dr. Woodward has stated that when a mallet is used upon mat gold it condenses very well. This is true; but I prefer, in using it, to employ hand-pressure with a broad instrument. I think we can do quite as well without the mallet.

I have examined this mat gold microscopically, and have failed to discover wherein it differs from other sponge gold. The material is exactly the same as Watt's crystal gold, as far as I have been able to observe. I think it will be found of value, but that it will always be difficult to make it perfect along the edges of cavities.

*Dr. McQuillen.*—I have been rather more fortunate in the use of crystal gold than Dr. Truman. Six or seven years ago Dr. Darby told me of fillings he had put in twenty years before, and I think that after seeing those fillings I used more crystal gold. I commenced its use at that time, and have been using it ever since. I do not think any of us would depend entirely upon this form of gold. I have been delighted with the margins, and I seldom see or hear of a filling of crystal gold leaking that I have put in. They have been very satisfactory, and since seeing those operations of Dr. Kirk's, at the last meeting, I have been using mat gold in the cases he describes,—large operations. I have been very much pleased with the result.

*Dr. Bonwill.*—Gold is a very interesting subject, yet I don't use as much of it as formerly.

It is a question whether we have any necessity for a new gold; whether that which we have is not all that is required for the use of any one in inserting a gold filling. I have used one kind, and still adhere to Abbey's. There are some objections I shall urge against the use of mat gold. The essayist says he is obliged to use hand-pressure as well as the mallet,—hand-pressure first, and the mallet afterwards,—until it is a perfect homogeneous mass. He can pound a great deal longer than he can with foil. He states that it will not do to overlap the wall of the tooth with it. It cannot be bevelled as gold-foil, as the edges will break. I don't know of a greater demand at present than for the perfection of gold-fillings by bevelling the cavities and carrying them over the edge,—over the wall of the cavity to prevent the wall from breaking. If I am to be restricted in the use of that gold in this direction, I would not use it.

The doctor then explained what he meant by bevelling.

*Dr. Peirce.*—The fillings placed in by Dr. Kirk were faithfully done and conditions observed that made it very practical. It is very admirable work. I think, as was said by several gentlemen, that sponge gold has a place in the office, and it can be used to very great advantage in many cases. And I also wish to emphasize what he said, that we cannot build out thin edges because it has not the tenacity of foil. If I have a large cavity,—a molar, for instance,—with a good wall, I can fill the cavity with mat gold in half the time I can with foil, and make an operation as serviceable as it is possible to make it. In that way it does us good service because it economizes time, but if we undertake to build out thin edges we are certain to make a failure of the operation.

*Dr. Guilford.*—I think mat gold differs from the other kinds of gold we have had in the finish of its crystals. How it is produced I do not know, but I believe it is by a method of deposit. In depositing copper, preparatory to making copper amalgam, we can have coarse or fine crystals, according to the quickness or slowness with which it is deposited on the electrode. I suppose they have a means of preparing it slowly and in finer crystalline form.

Sponge gold, in various forms, has been presented a number of times in the last twenty years. I remember very well a circumstance occurring several years ago. Dr. Magill was quite an advocate of the use of crystal gold. He used it extensively in his practice, and he called a number of us to see several mouths he had treated with fillings of this kind, and they were very beautifully done. A few years after, he wrote that he had abandoned it. He found that, after all, he had been more or less deceived by it, and returned to the kind of gold he had formerly used. I think, as others have remarked, that crystal gold has its place. There is no doubt we can do good work with it, and it is as good as any other kind of foil, under certain circumstances and conditions, but when we violate those conditions we have failure. I think if we have proper access to the cavity the filling can be made of crystal gold just as well as any other kind, if there has been sufficient experience in its use.

There is another objection to it, and I think it is a very serious one. It is recommended especially as a finish for fillings and for use at the cervical margins of the cavity. That is just the place I should object to employing it. It is very easily torn, broken, and powdered. Upon surface fillings we can get along with it, but at the margin it becomes brittle. I should never think of using it there, and have always found it unsatisfactory.

I have tested mat gold quite extensively in the last few months. I used it recently quite satisfactorily in a molar tooth that had a very large compound cavity, and next to it was a tooth filled with amalgam. I filled the posterior part with amalgam and upon that laid crystal mat gold. In that kind of filling I have found great difficulty in making it unite with the amalgam placed in the cavity, but with mat gold this was readily accomplished. In connection with phosphate of zinc I think it is very excellent.

It has its uses, yet at the same time I think it is a gold we dare not take liberties with, and must be careful in its manipulation.

Discussion closed.

The question for the evening was then taken up for discussion,—

"Should immediate root-fillings be practised while purulent conditions exist at the apex?" Opened by Dr. Peirce.

*Dr. C. N. Peirce.*—This question, as stated, implies that there is some expeditious and comparatively certain method of either removing, or else rendering innoxious the previously existing or recently acquired purulent condition at the end of the root, other than by the usual one, of either antiseptics, disenfectants, escharotics, astringents, alteratives, counter-irritants, desiccation, etc., and that without the use of these remedies, and in disregard of the septic conditions, the root-canal and crown-cavity can, immediately on the discovery of this previously estimated unfavorable pathological condition, be filled, and that sole reliance for subsequent comfort and success can be had through either systemic conditions or local recuperative power; the inference being that this accumulated pus and septic condition which is recognized, is immediately, on the tooth being filled, to be either transformed into nutrient pabulum, building-material, etc., or else taken up by the absorbents and carried off as a waste product. I must confess that I have not confidence in unaided natural processes accomplishing these results, yet the inquiry upon which this discussion is based may be answered in both the affirmative and the negative, the correctness of the answer depending entirely upon the condition of the surrounding structures which are present at the time the thus affected root is to be filled; for instance, an established fistula penetrating the process and overlying gum, through which the product of decomposition can find a ready means of exit; and after the canal has been placed in an antiseptic condition filling is not only pardonable, but under some circumstances desirable practice.

Through the fistula the apex of such a root could be reached and the purulent condition as readily overcome or corrected as by the application of remedies through the canal, while by the immediate filling after these prescribed conditions were secured the function of the tooth would be regained and the tissue at the apex protected from the danger of increased irritation by the ingress of foreign substances through the canal. On the other hand, if the conditions at the apex of the root are such as are frequently recognized, and the accumulated product kept at its minimum rate by an open canal, then to close this means of relief and discharge without first checking its accumulation would, in my judgment and experience, result in an inflammatory condition which would be anything but agreeable to the patient, suffering continuously, until relief was gained by either an artificial opening or through the pressure of accumulated pus, or absorption gave relief with a natural fistula. One or the other of these methods must be adopted to terminate the inflammation which will, with few exceptions, follow the abrupt closing of the only means of exit. These exceptions would of course be only in such cases as where the absorbents were sufficiently active as to overcome the accumulation of the purulent product; in that case it would be carried off with other waste material and the surrounding parts be thus freed or relieved from the septic influence.

There is another type where purulent conditions exist at the apex, and where, with a limited degree of safety, immediate root-filling may be practised. This is in teeth where the pulps had died without exposure. The cause of devitalization not being pertinent to the question, I shall not dwell upon it. Decomposition of pulp has followed, with only a very limited degree of irritation in the pericemental membrane at the apex of the root, but yet sufficient to establish a thickening of this root-covering, with some exudation, but both have been controlled by favorable systemic and local conditions, with activity of absorbents. The canal on being opened into is found filled with a yellowish fluid, but the parts around the apex have for months, probably, tolerated this condition, and would for months, and it may be years, longer. The opening into the crown and root has been made not to relieve suffering, but to prevent further discoloration of the crown. In this case, or in most of these cases, it is quite possible to cleanse the root, dry it as completely as possible, securing an absolute antiseptic condition so far as atmospheric germs are concerned, and fill at once with little or no danger of subsequent unfavorable conditions; and yet such treatment is not desirable practice.

Now, in these several conditions which have been indicated, where undoubtedly a purulent product exists, the remote but at one time quite general practice was to fill immediately, and then with a drill make an opening into the root-canal or pulp-chamber from just beneath the gum margin. This treatment has saved thousands of patients from discomfort while they yet had purulent conditions existing at the apex of root, but is it good practice except in some rare cases?

*Dr. Register.*—Since I have been using large quantities of hot and compressed air in connection with atomization my results in practice have been so different that I feel great good has been done,—certainly to my patients.

In regard to the question, I do not know whether it refers to indolent abscess or whether to putrescence in the pulp-chamber. If it is to putrescence from devitalized pulp, and there was no fistulous opening, I should make use first of a germicide, and then arrange the dam in place and use the hot air in large quantities, so that desiccation is thorough, then fill it while it is in that sponge-like condition. I think that immediate root-filling is indicated; certainly, from clinical experience. It has been my experience that it is good practice. It has been my impression that this carbonaceous matter that exists in a devitalized condition is the cause of the subsequent trouble with teeth of that character. Some have the idea that if the apex of the root be closed the trouble is avoided, but I think that is an error.

*Dr. Thomas.*—It may seem presumptuous for me to say anything in regard to root-filling where there has been a discharge either from the canal of the tooth or from a fistula. It has become a question in practice,—How long is a dentist justified in treating that root to stop the discharge? Experience has accumulated in capping exposed nerves. It was advocated that the nerve could be capped and the tooth made perfectly useful, yet it was a common thing for patients to come to me suffering from this treatment. In a case of pericementitis the patient may not come back, and possibly septic matter may have infiltrated along the tissues and formed a fistula. The tooth is treated, but there is an increase of inflammatory symptoms, infiltration, and another abscess. It may be treated again, but only after the formation of a fistula does the patient get relief; consequently there is no reason to visit a dentist, and probably there is no attempt to see one, and after a while it has gone so far that the process between it and the cuspid tooth has necrosed and the tooth must be removed. Is it good practice, or how long is it justifiable in a dentist to continue with such a tooth? and I would like, individually, to know whether you can make a tooth of that kind perfectly healthy and do away with the discharge; or how long would it remain so, or how much security can you give the patient that it will be a perfectly and permanently cured tooth? It grieves me to have a patient come for extraction with a filled tooth treated for abscess with a large fistula supposed to be cured. What can I do, and can I make it a perfectly well tooth? How long are you justified in treating it for that purpose?

*Dr. Truman.*—It does not appear to me that there is anything in this question. It is, Should you fill a tooth while it is in a purulent condition? No; of course you would not. That is the only answer I would give to that question. But in the broader sense, as I presume it is meant, will you do anything to a tooth which has pus in the canal? Dr. Peirce has taken up that point and handled it very well; but Dr. Thomas has brought up another,—Can we fill any tooth that has once had pus at the end of the root? I think you will agree with me, that pulpitis necessarily affects the pericementum. I do not think it is possible to have septic conditions in the pulp and not affect this membrane and increase further the development of pus; and wherever there is pus there must be destruction of tissue and a necrotic condition.

I have long entertained the idea that it was absolutely impossible to produce a healthy condition where pus has existed for a lengthened period at the end of the root. Immediate filling of such a tooth is, to my mind, impossible. We must change the conditions. If the root be necrosed, the dead tissue must be removed. If necrosis has not commenced, and the pulp is simply decomposed, it must be treated. Dr. Register says he accomplishes this by dry heat. I question this conclusion, as the heat required to destroy micro-organisms would destroy the tooth-tissues.

The whole territory of the dentine is filled with organic matter. It is in a decomposed state, and becomes a factor for future trouble. What is to be done with it? Years ago I recommended that it be coagulated, and that for this purpose chloride of zinc be used. It was said in answer that coagulation never would extend beyond the open mouths of the tubes. It has been demonstrated that it can be carried into the tooth. We can only approximate health in the treatment of many cases. When the destruction of the pericementum has been reached, we have arrived at a surgical operation,—cutting off the end of the root. Until that is done there will never be a healthy tooth in the mouth.

*Dr. Register.*—I rather hesitate to talk in reference to anything performed in my own practice. I offer the statement that I rarely at the present day have a fistula to treat. Only yesterday a lady came in to see me. She had a very bad molar and was in a very delicate condition. The central incisor had been treated for a number of years in the usual method. I operated on the tooth but once. It had a fistula and a gumboil at the end of it. I first washed it out with an atomizer, and after that by dilute sulphuric acid. I used this in about an eight-per-cent. solution, as a solvent to dissolve the carbonaceous matter that filled the tubulated structure of the dentine. I then followed this with Labaraque's solution. If it has a fistula it is given a treatment with acid of four-per-cent. solution.

I avoid using air intensely hot. The idea is to obtain thorough desiccation, and while in that condition to use some agent that will follow it up as a germicide. I have had a great deal of success with a preparation of from fifteen grains to an ounce of iodoform to an ounce of sulphuric ether. The odor is destroyed with oil of cinnamon. While the tooth is in this dry condition I saturate it with this liquid, and can immediately fill the canal with any preparation I prefer, either gold, or cotton saturated with phosphate of zinc or chloride of zinc.

*Dr. Guilford.*—I would like to know whether there is any one here who would have his teeth filled while there is a discharge of pus from the roots? It seems to me we would not be justified in doing for a patient what we would not want to have done to ourselves. It is evident, as far as the discussion has gone, that we have lost sight of the fact that there is such a thing as pyæmia. You remember how it carried off one of the Presidents of the United States. It is a serious matter, and I am surprised that it should be presented in this form. No one should think of filling while there are purulent conditions existing around the root.

Dr. Register stated that he did not mean to assert that he would fill a tooth having an indolent abscess. He desired it understood that he referred to a putrescent condition of the tooth-canal where there was no indication of a chronic abscess existing.

Discussion closed.

Incidents of practice were then taken up, and opened by Dr. Peirce, who said,—

I have a little matter here of some interest. There have been some questions in the journals in regard to the influence of abnormal conditions of the teeth in neuralgia and other disturbances. Some months ago a lady who had been present at one of my lectures on the influence of the teeth on facial neuralgia came to see me. She had worn an artificial denture for eight years. Upon examination of the mouth, I found on the left side a large tumor, for the accommodation of which the plate had been cut away. On passing a lance into the mass, an enamel surface was at once detected. On a free incision, opening it for the introduction of a pair of forceps beaks, an ordinary three-cusped, compressed-rooted third molar was readily removed. On further examination other teeth were recognized, which, upon removal with the forceps, proved to be three additional, supernumerary in character, united with apparently a membrane of connective tissue. On placing the four teeth together in the same relative position they had occupied in the cyst, the upper and larger one, the representative of the normal third molar, had been embedded with its crown towards the cheek. The next in size was closely adapted to a depression in the larger one, and at a slight angle to its vertical axis. The third in size was, in similar manner, fitted into a depression of the second in size, and in the same line deviating from its vertical axis; while the fourth in size, not much over a sixteenth of an inch in length, or rather between a sixteenth and an eighth, fitted into a well-marked cavity in the third or preceding in size and description. The crowns of these teeth all possessed multiple cusps; the largest one had, as above stated, the three cusps of an ordinary third molar, while the second in size had quite a pronounced central cusp encroaching upon a proximal surface, with three cusps on the other proximal side and one on each side of the more central cusp. The third tooth in size had four rounded cusps, with a deep sulci in the centre of the crown. The fourth and smallest of the group resembled more an inferior bicuspid, with the cusp on one side much more prominent than that on the other, both cusps, however, being divided by slight sulci. The point of most interest to the patient was that in the removal of these abnormal growths the suffering, which had been severe and of long duration, was entirely relieved.

*Dr. Darby.*—I saw an interesting case a few days ago. A lady came to me with a tumor inside the cheek as large as a thimble. In a very solemn way she said she wanted to consult me about something in her cheek, stating that her father had died with cancer, and, while she was not frightened, she thought this was of the same character. I examined it and found a top-shaped tumor protruding into her mouth just opposite the molar tooth of the anterior jaw. I asked her if she was not in the habit of drawing her cheek in by suction, and she replied she thought she was. I found she had lost the second molar, and the wisdom-tooth was in position, and that the suction had produced the tumor. I told her I did not think it was a cancer, and procuring a piece of floss-silk, I made a loop and passed it around it to shut off circulation, and told her to report to me if anything strange happened. Within the next few days I received a letter from her saying the cancer had dropped off into her mouth, and she was convinced it was not malignant.

J. ATKINSON MCKEE, D.D.S.,
*Editor Odontological Society of Pennsylvania.*

