# Correction

**Published:** 1893-03

**Authors:** Edwin T. Darby


### CORRECTION.

To the Editor:

Sir,—Will you kindly correct an error which appears in the February number of the Journal, page 127, in the discussion which followed Dr. Kirk's paper on "Crystal Mat Gold?"

I am in a measure responsible for this error, for I did not, it seems, confine myself strictly to the subject of the paper, but in the course of my remarks spoke of Watt's crystal gold. What I intended saying was that I had tried the *crystal mat gold* soon after it was introduced, and was not as favorably impressed with it as Dr. Kirk seemed to be.

I have used Watt's crystal gold more or less for twenty-five years, and consider it one of the best preparations of gold that we have ever had. The only adverse criticism which I made, or could make to it, is that it seems to deteriorate (lose its cohesive properties) more readily than other gold when exposed to the air. I should feel sorry, indeed, to have it understood that I was disappointed or displeased with Watt's crystal gold after all that I have spoken and written in favor of it.

Yours very truly,

Edwin T. Darby.

February 13, 1893.

